# Glycogen Synthase Kinase-3 Beta Expression Correlates With Worse Overall Survival in Non-Small Cell Lung Cancer—A Clinicopathological Series

**DOI:** 10.3389/fonc.2021.621050

**Published:** 2021-03-09

**Authors:** Marclesson Alves, Daniela de Paula Borges, Aline Kimberly, Francisco Martins Neto, Ana Claudia Oliveira, Juliana Cordeiro de Sousa, Cleto D. Nogueira, Benedito A. Carneiro, Fabio Tavora

**Affiliations:** ^1^ Department of Pathology, Federal University of Ceará, Fortaleza, Brazil; ^2^ Argos Pathology Laboratory, Department of Investigative Pathology, Fortaleza, Brazil; ^3^ Departments of Patholoy, Oncology and Thoracic Surgery, Messejana Heart and Lung Hospital, Fortaleza, Brazil; ^4^ Division of Hematology/Oncology, Lifespan Cancer Institute, The Warren Alpert Medical School of Brown University, Providence, RI, United States

**Keywords:** glycogen synthase kinase-3 beta (GSK-3 beta), lung cancer, non-small cell carcinoma, immunotherapy, programmed death-ligand 1 (PD-L1), phosphatase and tensin homolog deleted on chromosome 10 (PTEN)

## Abstract

**Background:**

Glycogen Synthase Kinase-3 beta (GSK-3β) regulates diverse cell functions including metabolic activity, signaling and structural proteins. GSK-3β phosphorylates target pro-oncogenes and regulates programmed cell death-ligand 1 (PD-L1). This study investigated the correlation between GSK-3β expression and clinically relevant molecular features of lung adenocarcinoma (PDL1 score, PTEN expression and driver mutations).

**Methods:**

We evaluated 95 lung cancer specimens from biopsies and surgical resections. Immunohistochemistry was performed to analyze the expression of GSK-3β, PTEN, and PDL1. Epidemiological data, molecular characteristics and staging were evaluated from medical records. The histologic classification was performed by an experienced pulmonary pathologist.

**Results:**

Most patients were female (52.6%) and the majority had a positive smoking history. The median age was 68.3 years, with individuals over 60 years accounting for 82.1%. The predominant histological subtype was adenocarcinoma (69.5%), followed by squamous cell carcinoma (20.0%). GSK-3β expression in tumors was cytoplasmic with a dotted pattern and perinuclear concentration, with associated membranous staining. Seven (7.3%) tumors had associated nuclear expression localization. Seventy-seven patients (81.1%) had advanced clinical-stage tumors. GSK-3β was positive in 75 tumors (78%) and GSK3-positive tumors tended to be diagnosed at advanced stages. Among stage III/IV tumors, 84% showed GSK3 positivity (p= 0.007). We identified a statistically significant association between GSK-3β and PTEN in the qualitative analysis (p 0.021); and when comparing PTEN to GSK-3β intensity 2+ (p 0.001) or 3+ expression (> 50%) – p 0.013. GSK-3β positive tumors with a high histological score had a worse overall survival.

**Conclusion:**

We identified the histological patterns of GSK-3β expression and evaluated its potential as marker for overall survival, establishing a simple histological score to measure the evaluated status in resected tissues. The use of GSK-3β expression as an immune response biomarker remains a challenge. Future studies will seek to explain the role of its interaction with PTEN.

## Introduction

Lung cancer represents a serious public health problem. In addition to its high incidence, this malignancy has the highest mortality rate worldwide (1). Glycogen synthase kinase-3 (GSK3) is a serine/threonine kinase, initially described as an ATP-Mg-dependent protein phosphatase (2), subdivided into two isoforms: GSK3 α and β (3). GSK3 was initially found to be related to various inflammatory processes, psychiatric disorders, neurodegenerative diseases, diabetes, cardiac dysfunction, autoimmune disorders, and more recently, it has been associated with cancer development (4, 5). It has been shown that GSK3 phosphorylates various components (TSC2, RICTOR, PTEN, and AKT) of the PI3K-AKT signaling network, an essential pathway for cell proliferation. Growth factors, cytokines, and chemokines are some of the signals that stimulate this process (6).

GSK3 is a central regulator of programmed cell death protein-1 (PD-1) expression, and GSK3 inhibition may downregulate PD-1 and enhance CD8+ cytolytic T cell (CTL) function (7). PD-1 and its ligand (PD-L1) are involved in the immune checkpoint pathway mechanism, of which activation promotes negative regulation of anti-tumor actions (8–11). In the same context, immunotherapy has become the new frontier to be explored in the cancer therapeutic arsenal, especially in lung cancer (12) (13). The level of PD-L1 expression measured by immunohistochemistry correlates with treatment response through the immune checkpoint inhibitor (14) (15). However, its quantification can be influenced by numerous variables, such as tumor heterogeneity, prior systemic therapy, radiation therapy, the molecular status of the neoplasia, type of sample analyzed, and different test methods (16).

**Figure 1:**
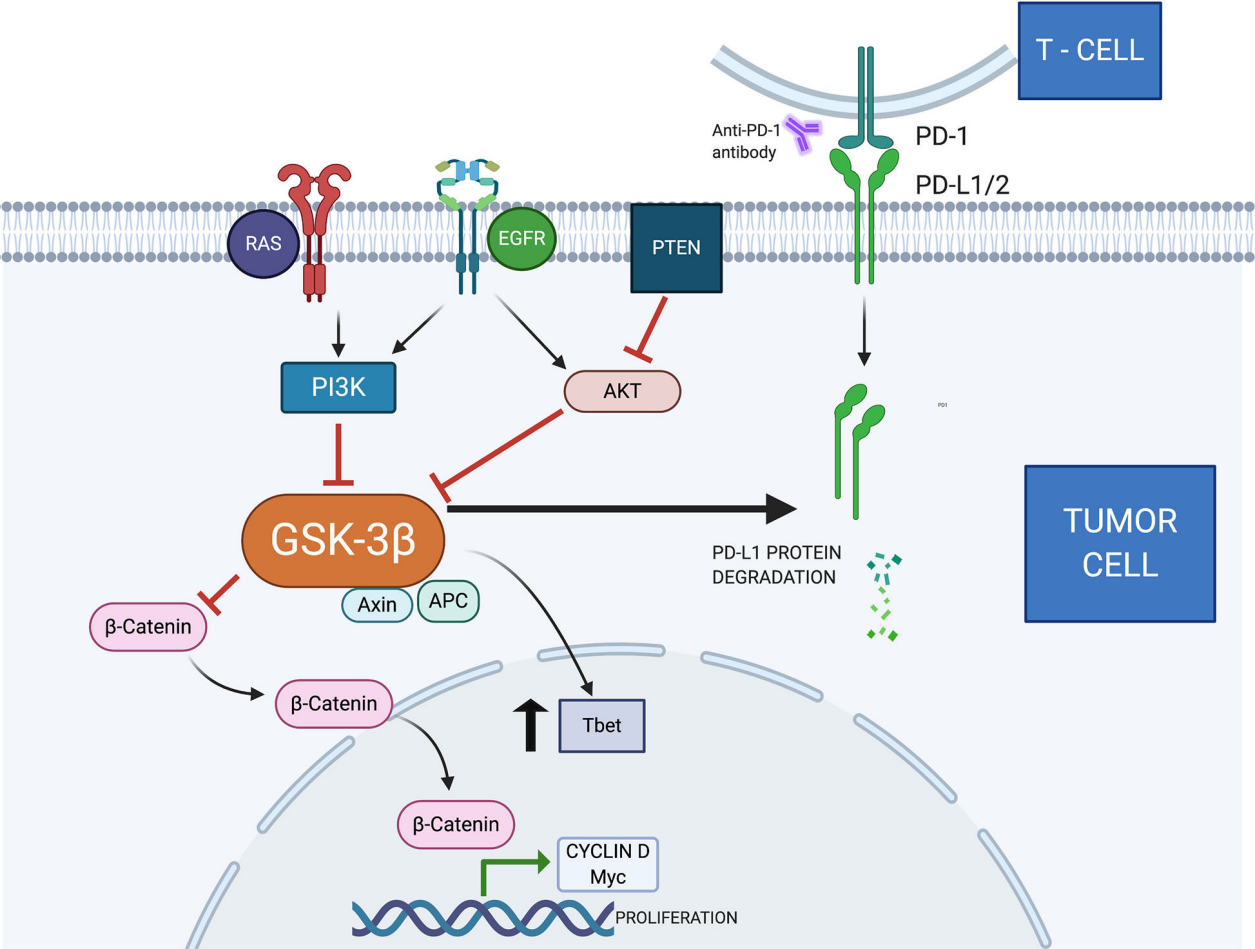
Several ligands on the cell surface stimulate the production of PIP3 (phosphatidylinositol trisphosphate) by PI3K. These molecules act as a substrate for protein activation, including AKT. This protein phosphorylates GSK3, inhibiting its function (6). The tumor suppressor gene PTEN blocks AKT activity by dephosphorylating PIP3 and PIK3 (21). In addition, Akt can activate the IκB complex (IKK), which phosphorylates IκB, becoming an important stimulus for NFκB (22). The NFkB is translocated to the nucleus, promoting the production of COX-2, an enzyme associated with angiogenesis, invasion, and metastasis (23). A complex consisting of AXIN, APC, and GSK3 is responsible for the destruction and negative regulation of B-catenin (24). The Wnt/β-catenin signaling pathway is related to cell proliferation, stem cell self-renewal, and cell differentiation (25). When active and in accumulation, B-catenin is translocated to the nucleus. Cyclin D1 and C-myc are oncogenes associated with cell proliferation and differentiation. Previous publications demonstrated they are target genes for the Wnt/B-catenin signaling pathway (26). T-box transcription factor protein 21 (TBX21)—also known as Tbet—is present in tumor cells, causing local immune response dysregulation and carcinogenesis (27). GSK3 inhibition promotes increased Tbet transcription (28). Previous reports demonstrated that GSK3 can cause PDL1 degradation by the proteasome pathway (29). GSK3, Glycogen Synthase Kinase-3; PI3K, Phosphatidylinositol-3-kinase; AKT, protein kinase B; MAPK, Mitogen-Activated Protein Kinase; APC, Adenomatous polyposis coli; Axin, Axis inhibitor; NFκB, Nuclear factor-κ light chain enhancer of activated B cells; PTEN, Phosphatase and tensin homolog; COX-2, Cyclooxygenase-2; EGFR, Epidermal growth factor receptor; MyC, Myelocytomatosis—proto oncogene; Tbet, transcription factor T-box expressed in T cells; RAS, Rat sarcoma—oncogene; PD-L1, Programmed death-ligand 1.

PTEN negatively regulates the PI3K/AKT pathway, playing an important role in intracellular growth, leading to decreased phosphorylation of AKT substrates, including BAD (BCL-2- associated agonist of cell death) and GSK-3 (17, 18). PTEN functions as a tumor suppressor gene that antagonizes PI3K activity (19). Several mechanisms can identify PTEN loss, such as mutations, deletions, absence of protein expression, and promoter methylation. In lung tumors, the absence of immunohistochemical expression is seen in 30%–50% of cases (20). (**Figure 1**).

Considering these interactions with components of tumor growth pathways, GSK-3β has become a critical molecule to be used in the fight against cancer and the development of new drugs (30). In the search for the improvement and innovation in biomarkers, recognizing its potential to influence carcinogenesis, and considering its role in other inflammatory pathologies, our study analyzed the GSK-3β expression in lung cancer and its correlation with PDL1, the best predictor of response to immunotherapy used in clinical practice.

## Materials and Methods

### Patient and Tissue Selection

We sequentially selected 95 patients diagnosed with lung cancer between 2013 and 2019 in the State of Ceará, using available material (paraffin blocks) and follow-up information from an approximate cohort of 450 newly diagnosed patients from the same period. All patients had clinically and pathologically confirmed tumors as primary lung cancer. The Ethics Review Board reviewed and approved this research study. Cases were diagnosed by a single Thoracic Pathologist based on the current WHO criteria (31, 32).The inclusion criteria consisted of non-small cell lung cancer with enough tissue sample (paraffin blocks with tissue containing more than 100 tumor cells after all recuts) and availability for additional immunohistochemical studies. All patients with secondary tumors of the lung were excluded from the study. Epidemiology and clinicopathological characters (such as gender, age, smoking status, comorbidities, TNM stage, survival, molecular mutations) were obtained from medical records or by contacting the patients.

Tumor necrosis was defined as any amount of necrotic tumor, as an area of increased eosinophilia without tumor cell nuclei, only the shadow of membranous tumor cells or with nuclear shrinkage or fragmentation.

### Immunohistochemistry

Immunohistochemistry (IHC) was performed on histological sections of tumors obtained from biopsies or resections. The sections were cut and mounted on electrically charged glass slides. GSK-3β (Cell Signaling, clone 27C10) and PTEN (Clone SP218; Roche Diagnostics Limited, Burgess Hill, UK) IHC analyses were performed on the Ventana^®^ platform (BenchMark ULTRA IHC/ISH Staining Module, Ventana Medical Systems, Tucson, AZ). The PD-L1 IHC analysis (22C3 pharmDx, Agilent, clone 22c3) was recovered using PT-Link (Dako PT100), followed by target recovery with EnVision ™ FLEX pH 6.0 buffer, using the Agilent Technologies^®^, USA visualization system in Autostainer Link 48^®^ equipment.

GSK-3ß was recorded according to the cytoplasmic and membranous staining intensity as 0 (negative), 1+(weak), 2+ (moderate), and 3+ (strong), and the percentage of stained tumor cells. A final histological score was established ([% of weak staining × 1] + [% of moderate staining × 2] + [% of strong staining × 3]) to determine the overall percentage of GSK-3ß positivity across the entire stained tumor sample, yielding a range from 0 to 300. Any tumor with a score >200 was considered as showing high GSK-3ß expression.

PD-L1 was assessed as membranous positivity in tumor cells, following the Tumor Proportion Score (TPS) as practiced currently (33). We tried different cutoff points to reflect clinical scenarios: <1%, 1%–49%, ≥50%. In investigating the correlation with PTEN, we use yet another method of quantification, characterizing PD-L1 as positive or negative expression if tumor cells had a positivity above 1%.

PTEN protein was considered lost if the cytoplasmic and nuclear staining intensity was markedly decreased or entirely negative across >10% of tumor cells when compared to surrounding benign tissue, which provides internal positive controls. Other molecular data (*EGFR*, *ALK*, *BRAF*, and *KRAS* status) were retrieved from medical records, and correlation with the protein expression findings was attempted.

### Statistical Analysis

Patients were followed for up to 68.3 months (mean follow-up of 18.8 months, SD 14.3 months). Distant metastasis was defined as recurrence at any site other than the abovementioned ones and was confirmed by imaging studies and histopathological evidence, when necessary. Overall survival (OS) was defined as the interval between the initial diagnosis and death (event) or the last follow-up date.

Statistical evaluation was performed by Stata® version 13 statistical software (StataCorp LP, College Station, TX, USA). Initially, a descriptive analysis of the study population variables was performed, calculating absolute and relative frequencies. Subsequently, bivariate analysis was performed using Pearson’s or Fisher’s chi-square test (when expected values of the contingency table cell were <5), with their respective 95% confidence intervals and statistical significance (p <0.05).

Also, multiple regression was run to predict the overall survival based on the variables age, gender, smoking, histology, treatment (QT), clinic stage, EGFR, PD-L1, and GSK3β expression. The assumptions of linearity, independence of errors, homoscedasticity, unusual points, and normality of residuals were tested using SPSS 24.0 (SPSS Inc., Chicago, IL, USA).

## Results

### Patient Demographics and Treatment

We evaluated 95 patients with primary lung cancer (90 samples from lung parenchyma and five from pleural tissue). Most patients were female (52.6%), and the majority had a positive smoking history (77.7% of the males and 52% of females). The median age at diagnosis was 68.3 years (range, 32.1–94.9 years), with individuals over 60 years accounting for 82.1%. All data regarding the clinical and pathological factors are presented in **Table 1**.

**Table 1:** Analysis of clinico-pathologic characteristics of studied population by GSK-3β expression, with bivariate and multivariate analysis.

		GSK		Bivariate			Multivariate		
	Total	Negative	Positive	RP	CI95%	P-value	RP*	CI95%*	P-value*
	N (%)	N (%)	N (%)						
Gender									
Female	50 (52.6)	8 (16.0)	42 (84.0)	1.14	0.92–1.42	0.203			
Male	45 (47.4)	12 (26.7)	33 (73.3)	Ref					
Ethnic group									
Caucasian	42 (44.2)	6 (14.3)	36 (85.7)	Ref			0.73	0.41–1.27	0.265
African-Brazilian	2 (2.1)	2 (100)	–						
Multiracial	51 (53.7)	12 (23.5)	39 (76.5)	0.89	0.73–1.08	0.261			
Age (years)									
<60	17 (17.9)	4 (23.5)	13 (76.5)	Ref					
≥60	78 (82.1)	16 (20.5)	62 (79.5)	1.04	0.78–1.38	0.782			
Smoking									
No	34 (35.8)	4 (11.8)	30 (88.2)	Ref			0.43	0.12–1.47	0.178
Yes	61 (64.2)	16 (26.2)	45 (73.8)	0.83	0.69–1.01	0.097			
Site (lobe)									
Inferior Right	17 (17.9)	5 (29.4)	12 (70.6)	Ref					
Inferior Left	18 (18.9)	4 (22.2)	14 (77.8)	1.10	0.74–1.63	0.628			
Middle	5 (5.3)		5 (100)						
Superior Right	26 (27.4)	6 (23.1)	20 (76.9)	1.09	0.75–1.58	0.642			
Superior Left	24 (25.3)	5 (20.8)	19 (79.2)	1.12	0.77–1.62	0.529			
Histology									
Adenocarcinoma	66 (69.5)	13 (19.7)	53 (80.3)	1.34	0.79–2.25	0.151			
Squamous Cell Carcinoma	19 (20.0)	3 (158)	16 (84.2)	1.40	0.82–2.41	0.148			
Other	10 (10.5)	4 (40.0)	6 (60.0)	Ref					
Adenocarcinoma subtype									
Acinar	26 (39.4)	3 (11.5)	23 (88.5)	2.06	0.87–4.91	0.009			
Lepidic	7 (10.6)	4 (57.1)	3 (42.9)	Ref					
Minimally invasive	1 (1.5)		1 (100)						
Invasive mucinous	1 (1.5)		1 (100)						
Solid	25 (37.9)	5 (20.0)	20 (80.0)	1.87	0.78–4.49	0.053			
T (primary tumor)									
1 + 2	51 (56.7)	9 (17.6)	42 (82.4)	1.07	0.86–1.32	0.523			
3 + 4	39 (43.3)	9 (23.1)	30 (76.9)	Ref					
N (regional lymph node)									
0 + 1	41 (45.1)	11 (26.8)	30 (73.2)	Ref					
2 + 3	50 (54.9)	8 (16.0)	42 (84.0)	1.15	0.92–1.43	0.206			
TNM Stage									
I + II	18 (18.9)	8 (44.4)	10 (55.6)	Ref			4.59	1.43–14.72	0.010
III + IV	77 (81.1)	12 (15.6)	65 (84.4)	1.51	0.99–2.32	0.007			
EGFR									
Inconclusive	2 (2.1)	1 (50.0)	1 (50.0)	Ref					
Negative	45 (47.4)	10 (22.2)	35 (77.8)	1.55	0.38–6.27	0.364			
Positive	20 (21.1)	2 (10.0)	18 (90.0)	1.80	0.45–7.25	0.116			
ALK									
Negative	55 (57.9)	11 (20.0)	44 (80.0)	Ref					
Positive	6 (6.3)	1 (16.7)	5 (83.3)	1.04	0.71–1.52	0.845			
PDL1									
Negative	62 (65.3)	14 (22.6)	48 (77.4)	Ref					
Positive	29 (30.5)	5 (17.2)	24 (82.8)	1.07	0.86–1.32	0.559			
PDL1 score									
<1%	61 (64.2)	14 (22.9)	47 (77.1)						
1-49%	17 (17.9)	3 (17.6)	14 (82.4)	1.07	0.82–1.38	0.639			
≥50%	13 (13.7)	2 (15.4)	11 (84.6)	1.09	0.84–1.44	0.547			
ROS1									
Uncertain	1 (1.1)		1 (100)						
Inconclusive	2 (2.1)	1 (50.0)	1 (50.0)	Ref					
Negative	26 (27.4)	4 (15.4)	22 (84.6)	1.69	0.42–6.38	0.281			
BRAF									
Negative	15 (15.8)	4 (26.7)	11 (13.3)			0.551			
Positive	1 (1.1)		1 (100)						

Seventy-seven patients (81.1%) had advanced clinical-stage tumors at diagnosis (Stages III–IV). Treatment consisted of systemic therapy (including cytotoxic therapy, immunotherapy, or both) in 70.5% patients, lobectomy in 25.2% patients, segmentectomy in 2.1%, and pneumonectomy in 2.1%. Patients following chemotherapy standard protocol represented 85.5% of the cohort, with an additional 25.5% undergoing anti-PD1 immunotherapy regimens. Tyrosine kinase inhibitors corresponded to treatment in 20.2% of cases. Antiangiogenics were found as part of therapy in only 10% of patients. Radiation therapy was administered as a therapeutic modality in 51.1% of patients. It was used to control bone pain, definitive therapy concomitant with chemotherapy, or finally approach brain metastasis. Tumors were in the right upper lobe in 27.4%, followed by the left superior in 25.3%, left inferior in 18.9%, right inferior in 17.9%, and the middle lobe in 5.3%.

### Pathological Classification

The predominant histological subtype was adenocarcinoma (69.5%) followed by squamous cell carcinoma (20.0%). The remainder were unclassifiable non-small cell carcinoma with characteristics of large-cell carcinoma (WHO recommendation). Invasive adenocarcinomas with predominant acinar and solid patterns of growth were the most often identified (39.4% and 37.9%, respectively). There were seven lepidic-predominant (10.6% of adenocarcinomas), one mucinous, and one minimally invasive adenocarcinoma (1.5% each). Most squamous cell carcinomas were moderately differentiated non-keratinizing, tumors, showing the usual histology. Non-small cell, not otherwise specified carcinomas, did not show glandular, squamous or neuroendocrine differentiation either by light microscopy morphology or immunohistochemistry. Tumor necrosis was present at least focally in 40% of cases (38 cases) and showed a strong correlation with solid growth pattern [high-grade, (R=0.335, p=0.012)].

### GSK-3 Beta Protein Expression

**Figure 2:**
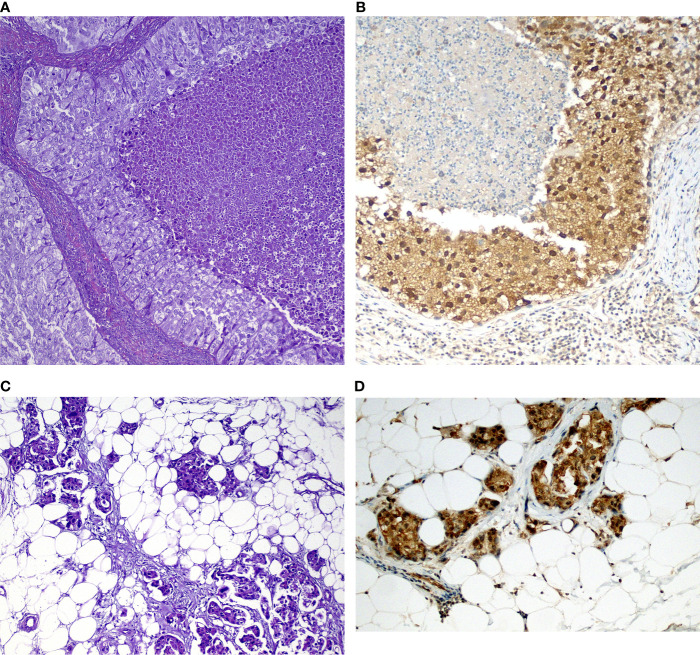
Examples of tumors and corresponding immunohistochemical stains in **(A)** Solid-predominant adenocarcinoma with tumor necrosis (center) and over atypia; **(B)** GSK-3β expression in the tumor above showing strong (3+) positivity in both nuclei and cytoplasm. Note weak GSK-3β expression in small lymphocytes (arrowheads). **(C)** Acinar adenocarcinoma metastatic to the parietal pleura (adipose tissue) showing **(D)** cytoplasmic staining in tumor cells.

**Figure 3:**
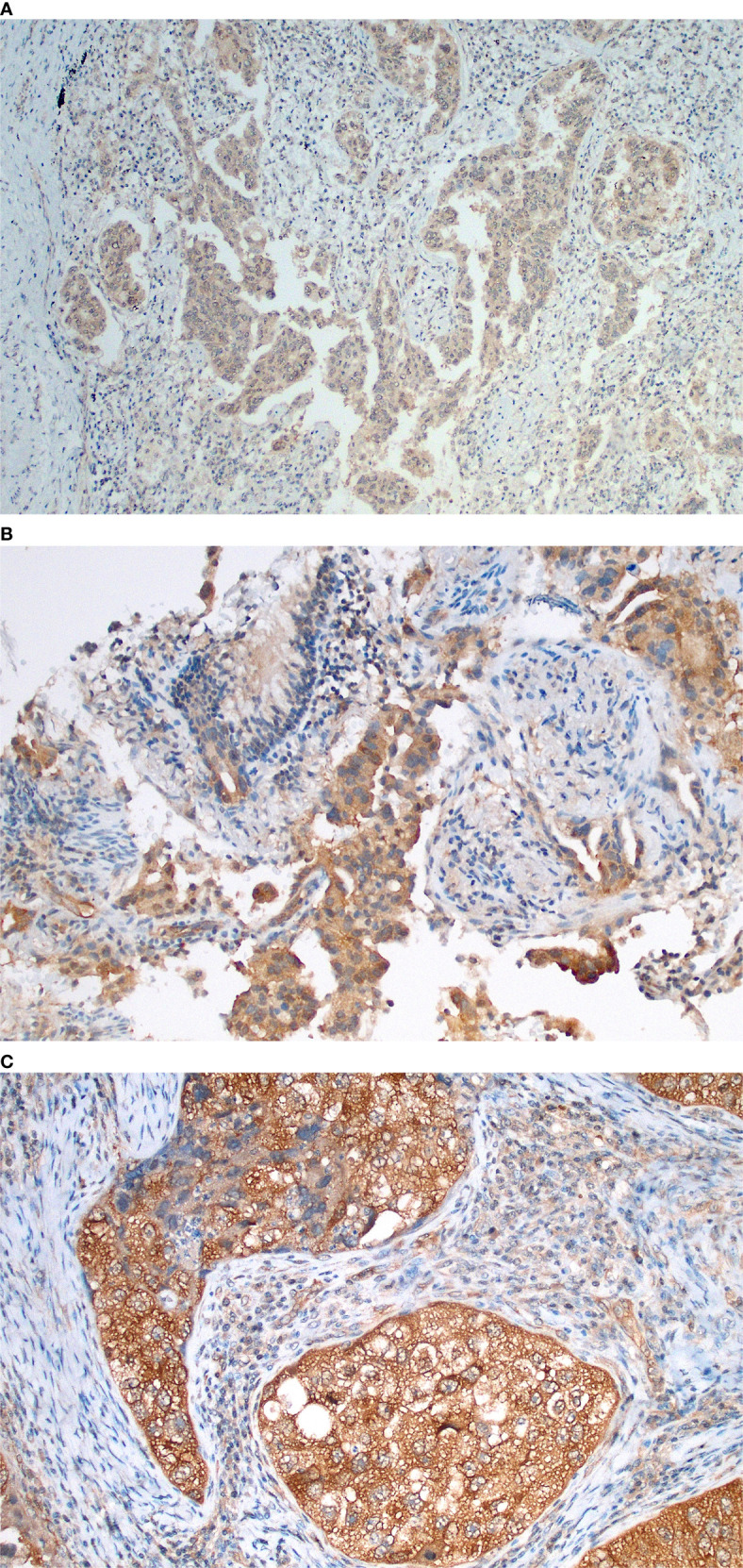
Immunohistochemical findings of GSK-3β in lung adenocarcinomas, demonstrating semi-quantitative score examples and showing distinct membranous and cytoplasm expression. **(A)** GSK-3β weak (1+) expression in acinar predominant lung adenocarcinoma; **(B)** Moderate expression (2+) in acinar predominant lung adenocarcinoma evaluated in a transbronchial biopsy sample; **(C)** Strong expression in a solid-predominant adenocarcinoma with focal nuclear localization and association with tumor necrosis.

GSK-3β was identified in 75 tumors (78%), being considered high in 17 cases (22.7%) and low in 58 cases (77.3%) (**Figures 2** and **3**). There was no difference in GSK-3β positivity when comparing gender, age, or smoking history. GSK-3β-positive tumors were more prevalent in advanced-stage tumors. Among stage III/IV tumors, 84% showed GSK3 positivity, in contrast with 55.6% of stage I/II tumors, with statistical significance in the univariate and multivariate analyses (p = 0.007) (**Table 2**). Interestingly, when evaluating by size alone, we identified a greater number of patients with GSK-3β expression in T1/T2 tumors (95%CI 0.86–1.32, p = 0.523), compared to T3/T4 cancers, but without statistical significance.

**Table 2:** Bivariate analysis and correlations of GSK-3β expression and PTEN status in lung tumors.

	Total n (%)	PTEN		Bivariate		
		Negative	Positive	RP	CI95%	P-value
		N (%)	N (%)			
GSK-3β						
Negative	7 (25.9)	6 (85.7)	1 (14.3)	Ref		
Positive	20(74.1)	7 (35.0)	13(65.0)	4.55	0.72–28.73	0.021
GSK Intensity						
0	7 (25.9)	6 (85.7)	1 (14.3)	Ref		
1	6 (22.2)	4 (66.7)	2 (33.3)	2.33	0.27–19.8	0.416
2	12(44.4)	1 (8.3)	11(91.7)	6.42	1.04–39.7	0.001
3	2 (7.4)	2 (100)				
GSK score						
<1%	7 (25.9)	6 (85.7)	1 (14.3)	Ref		
1%–49%	6 (22.2)	3 (50.0)	3 (50.0)	3.50	0.48–25.4	0.164
≥50%	14(51.8)	4 (28.6)	10(71.4)	5.00	0.79–31.6	0.013
PDL1						
Negative	20(76.9)	8 (40.0)	12(60.0)	Ref		
Positive	6 (23.1)	4 (66.7)	2 (33.3)	0.55	0.17–1.82	0.251
PDL1 score						
<10%	22(81.5)	10 (45.4)	12(54.6)	Ref		
≥10%	5 (18.5)	3 (60.0)	2 (40.0)	0.73	0.23–2.29	0.557
PDL1 analysis						
<1%	21(77.8)	9 (42.9)	12(57.1)	1.42	0.46–4.45	0.489
1%–49%	5 (18.5)	3 (60.0)	2 (40.0)	Ref		
≥50%	1 (3.7)	1 (100.0)				

We found no statistically significant differences regarding GSK-3β and driver mutations, such as *EGFR*, *ALK*, *ROS1*, or *BRAF* (**Table 1**). Only 11 patients were tested for KRAS mutations. Three cases were KRAS mutated (27.2%), while eight were KRAS wild type (72.8%). There was no direct correlation between GSK-3β expression and PD-L1 positivity or PD-L1 percentage. When considering the PDL1 score most often used in clinical trials and treatment guidelines (negative, 1%–49% and > 50%), no correlation was found. The data is summarized in **Table 1**. Tumor necrosis was also found to be statistically significant with PD-L1 expression regardless of PD-L1 cutoff levels (p<0.001), but there was no correlation between tumor necrosis and GSK-3β expression in the cohort.

Multivariate logistic regression analysis further showed that overall survival was related to EGFR gene mutation (p=0.023), final clinical staging (p=0.033) and GSK-3β expression (p=0.035). These results indicate that the expression of GSK-3β could be potentially a marker of overall prognosis independent of driver mutation status and is correlated with smoking status and clinical stage (**Table 3**).

**Table 3:** Multiple regression to predict overall survival based on clinic variables and GSK-3β expression.

Variables	Unstandardized Coefficients		Standardized Coefficients	t	Sig.
	B	Std. Error	Beta		
Death (Constant)	3.994	0.917		4.353	0.000
Age	-0.005	0.006	-0.126	-0.895	0.376
Gender	-0.149	0.148	-0.161	-1.010	0.319
Smoking	0.147	0.146	0.155	1.006	0.321
Histology	0.111	0.150	0.119	0.741	0.463
Treatment	-0.187	0.261	-0.123	-0.719	0.477
PDL1	-0.200	0.142	-0.203	-1.411	0.167
EGFR	-0.453	0.192	-0.446	-2.361	**0.023**
Clinic stage	-0.185	0.084	-0.354	-2.207	**0.033**
GSK-3β	-0.375	0.171	-0.328	-2.189	**0.035**

Bold text indicates significant differences (p < 0.05).

Of note, we identified a statistical correlation between GSK3 and PTEN (95% CI 0.72–28.73, p = 0.021) with the greater number of PTEN positivity in cases where GSK3 intensity score was = 2 (95%CI 1.04–39.7, p = 0.001) (**Table 2**).

### Survival Analysis

**Figure 4:**
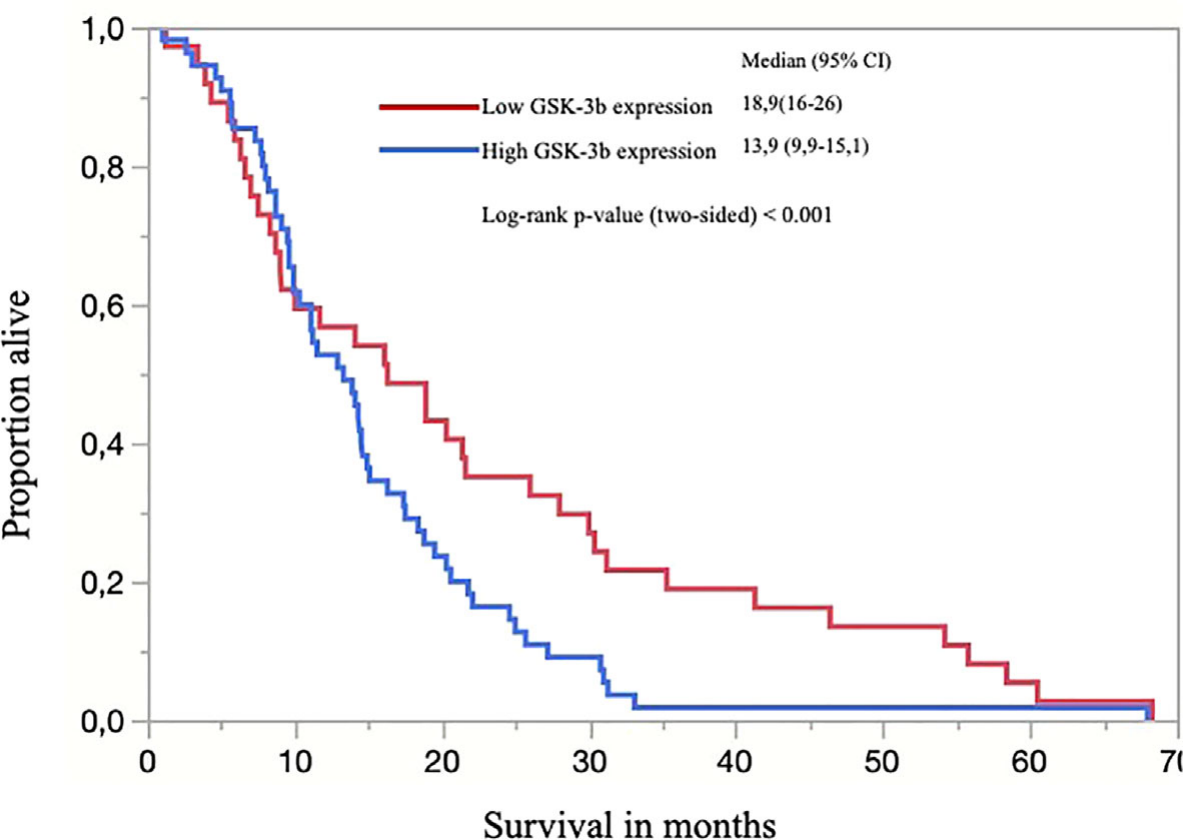
Kaplan-Meier curve of overall survival (OS) for all patients separated by low (score <200) and high expression of GSK-3β and; the censored events were defined for OS as time to the last known date alive before analysis or date of death.

Patients were followed for up to 68.3 months (median 14.1, range 1.0–68.3). Patients with adenocarcinomas survived on average 19.6 months and patients with squamous cell carcinomas, 14.5 months. Solid-predominant tumors had a poor survival rate when compared to the remainder (p<0.01). There was a significant difference when comparing the survival of patients with high GSK expression with patients with low or absent GSK (p<0.0062; **Figure 4**, Kaplan-Meier). We also found a significant difference in survival in patients presenting with higher stage and lymph node metastasis, as expected (p=0.010). There was no statistical difference regarding survival when comparing PDL1, ALK, or EGFR status alone or in combination with GSK-3β. Patients with PTEN positivity had a better overall survival (p=0.026).

## Discussion

Immune manipulation is a chapter of extensive research in oncology. Knowledge about biomarkers predicts better outcomes and results. Our article studied GSK-3 beta protein expression, tumor prognosis, and possible interactions in non-small cell lung cancer. The high expression of GSK-3 was correlated to a worse prognosis, underscoring the importance of studies hypothesizing that blocking GSK can be a potential therapeutic target in non-small lung cancer. After understanding their intracellular interactions, GSK3 inhibitors have been researched in the treatment of cancer (34). Many types of drugs have been developed (35) and are being tested in pre-clinical and phase I studies, with promising results (36).

We identified a greater number of advanced-stage III/IV tumors in patients with high GSK3 expression (p=0.007). This translates into a possible more aggressive biological component in this population, or even an association with different genotypes, since higher tumor mutation burden (TMB) has been associated with advanced lung tumors. In contrast, recent studies have shown that tumors with high TMB may respond better to nivolumab and pembrolizumab (37). Somatic mutations can produce neoantigens, immunogenic peptides that activate the immune response. TMB and PDL1 have the same clinical applicability. However, TMB represents a complement and not a substitute for PDL1 (38). Another interesting finding in the current study is the correlation of tumor necrosis and PD-L1 expression. These are in accordance to other studies that showed tumors with higher PD-L1 expression with a greater tendency to necrosis, a more aggressive tumor phenotype and higher proliferation rate (39, 40).

Tumor cells have developed several mechanisms to escape immune surveillance. GSK3 stands out in the regulation of the PD-1/PD-L1 inhibitory checkpoint. This immune activation process can occur through PD-1 interaction or modulation in the tumor molecule PD-L1. Li et al. demonstrated that GSK-3β correlates to PD-L1 and induces phosphorylation-dependent degradation of PD-L1 by b-TrCP (41). The inhibition of GSK3 also acts to promote PD1 downregulation. When associated with anti-PD1 or anti-PDL1 block, it enhances the cytotoxic capacity of T cells (42). Taylor et al. demonstrated that GSK-3 participates in the regulation of PD-1 transcription. GSK-3 inhibition increases Tbet activity, reducing PD-1 transcription, with a further intensification of T lymphocyte cytolytic action (28). Despite the theoretical rationale supporting this association, we did not identify a relationship between PDL1 and GSK-3β scores in our cohort. A recent publication about the positivity of PDL1 in the same region of northeastern Brazil showed that 59.5% of patients were PDL1 negative (43).

Matsuo et al. studied the importance of AKT, mTOR, and GSK3 in the occurrence of lymph node involvement in oral cavity squamous cell carcinomas. Elevated expression of GSK3 and pGSK3-βSer9 was associated with metastasis in cervical lymph nodes (p = 0.004 and p = 0.03) (44) and advanced stages (cTNM), suggesting the relationship of GSK3 with tumor invasion and metastases. In our study, of the few squamous cell carcinomas studied, the expression score of GSK3 was more evident in poorly differentiated tumors and advanced stages. Blocking GSK-3β reduced cell proliferation, stimulated apoptosis, maintained cells in the GO/G1 phase and increased cell invasion. A study published by Zeng et al. focused on the association between GSK3 and survival. The positive expression of GSK3 by immunohistochemistry was also related to a worse prognosis (45). Therefore, the relationship between GSK3 and direct survival has been hypothesized.

Cigarette exposure corresponds to a cluster of toxic substances that promote damage to alveolar cells (46). The mechanisms involved are still not fully understood. Besides, it induces beta-catenin accumulation (47). Nagahori et al. analyzed a polymorphism of the GSK3 gene and its connection with smoking habits. In a cohort of 384 patients, rs334558 was associated with smoking in genotype and allelic frequency (48). Numajiri M et al. also published an article describing the relationship between a polymorphism of the *GSK3* gene and its relationship with nicotine dependence (49). Despite the possible inhibition of GSK3 by smoking, we found no statistical basis for this association in our population (p=0,097).

Anaplastic lymphoma kinase (ALK) is a tyrosine kinase with altered expression in several tumors. In lung cancer, ALK-EML4 rearrangement occurs in about 3%–5% of cases (50), allowing the use of target drugs and achieving better survival results (51). Malagon et al. analyzed the relationship between ALK and GSK3 in neuroblastomas and neural crest cells. They suggest a positive regulation of GSK3 *via* ALK tyrosine kinase (52). McDonnell SRP et al. studied anaplastic large-cell lymphoma, a pediatric disease, in which the NPM-ALK alteration is present in 70%–80% of cases. They revealed that ALK activity acts on the phosphorylation of S9-GSK-3 *via* PI3K/AKT, making GSK3 an important regulator of ALK carcinogenesis (53).

EGFR is a surface receptor, a HER family component, also known as erbB1, HER1, or even as EGFR (54). Its activation triggers intracellular cascades, promoting cell growth and oncogenesis (55). It is especially important in lung tumors, acting as a predictor of response to tyrosine kinase inhibitors (56). Fitzgerald et al. studied the role of EGFR in the intracellular signaling pathways of pancreatic tumors. EGFR activates the Ras/Raf/MEK/ERK pathway. Consequently, it stimulates the ETS transcription factor that binds to the GSK-3beta promoter and induces the expression of GSK-3 and IKK. Subsequently, this regulates the production of NF-kappa-B, leading to gene transcription and cell proliferation (57). This exemplifies a possible relationship between EGFR mutations and GSK3 expression.

In the current study, an additional analysis in a subset of 27 resection samples demonstrated a statistically significant association between GSK-3β and PTEN analysis by immunohistochemistry (p = 0.021). This relationship was more evident in cases with strong GSK-3β expression or the expression in > 50% of the tumor. Zhengyu H et al. demonstrated that PTEN overexpression reduces fibroblast proliferation by inhibiting the PI3K/AKT/GSK3 pathway (26). Gao C. et al. showed by measuring the levels of GSK-3β, PTEN, and AKT in breast tumor cell lines, that the PTEN/PI3K/AKT pathway can be regulated by GSK3 (58). PTEN inactivation is associated with lower survival and resistance to systemic treatment (59). These findings underscore the importance of GSK activation in tumor survival and growth regulation (60).

Although previously identified as a tumor suppressor, PTEN is not currently used in clinical practice as a lung cancer biomarker. While active PTEN has anti-tumor action, resulting in better outcomes (18, 61, 62). This is in contrast to our results, which shows a positive correlation. Further research involving GSK3 and PTEN blockers in lung cancer is necessary for better clarification and therapeutic use to manipulate these intracellular checkpoints.

In conclusion, GSK-3β is a molecule involved in multiple signaling pathways. Positive tumor expression was associated with a more advanced tumor stage and worse overall survival in lung cancer. To the best of our knowledge, this is the first study to identify the expression of GSK-3β as a potential marker for overall survival and establish a simple histological score to be measured in resected tissues. The use of GSK-3β expression as an immune response biomarker remains a challenge. Future studies will seek to explain the role of its interaction with PTEN.

## Data Availability Statement

The original contributions presented in the study are included in the article/supplementary material. Further inquiries can be directed to the corresponding author.

## Ethics Statement

The studies involving human participants were reviewed and approved by Federal University of Ceará. Written informed consent for participation was not required for this study in accordance with the national legislation and the institutional requirements.

## Author Contributions

MA, BC, and FT designed the study. DB, AK, and JS performed all molecular studies and immunohistochemical studies. MA and FM reviewed patients charts and follow-up information. Slides were evaluated by pathologists FT, CN, and AO. MA, FT, and BC wrote and reviewed the final manuscript. FT is the lead investigator. All authors contributed to the article and approved the submitted version.

## Conflict of Interest

The authors declare that the research was conducted in the absence of any commercial or financial relationships that could be construed as a potential conflict of interest.
